# Slicing and Dicing: Optimal Coarse-Grained Representation to Preserve Molecular Kinetics

**DOI:** 10.1021/acscentsci.2c01200

**Published:** 2023-01-17

**Authors:** Wangfei Yang, Clark Templeton, David Rosenberger, Andreas Bittracher, Feliks Nüske, Frank Noé, Cecilia Clementi

**Affiliations:** † Center for Theoretical Biological Physics, Rice University, Houston, Texas77005, United States; ‡ Graduate Program in Systems, Synthetic and Physical Biology, Rice University, Houston, Texas77005, United States; ¶ Department of Physics, Freie Universität Berlin, Arnimallee 12, 14195Berlin, Germany; § Department of Mathematics and Computer Science, Freie Universität Berlin, Arnimallee 12, 14195Berlin, Germany; ∥ Max Planck Institute for Dynamics of Complex Technical Systems, Sandtorstrasse 1, 39106Magdeburg, Germany; ⊥ Department of Chemistry, Rice University, Houston, Texas77005, United States; # Department of Physics, Rice University, Houston, Texas77005, United States

## Abstract

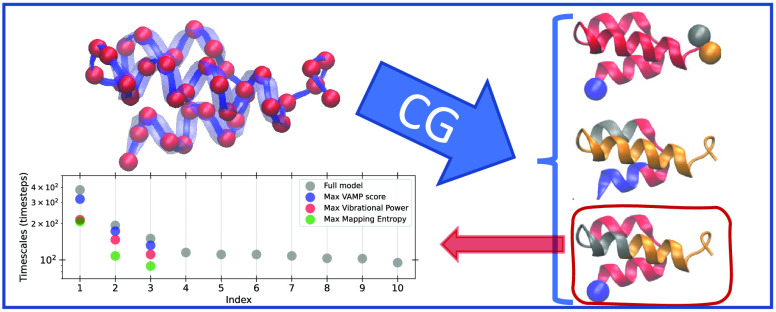

The aim of molecular coarse-graining approaches is to recover relevant physical properties of the molecular system via a lower-resolution model that can be more efficiently simulated. Ideally, the lower resolution still accounts for the degrees of freedom necessary to recover the correct physical behavior. The selection of these degrees of freedom has often relied on the scientist’s chemical and physical intuition. In this article, we make the argument that in soft matter contexts desirable coarse-grained models accurately reproduce the long-time dynamics of a system by correctly capturing the rare-event transitions. We propose a bottom-up coarse-graining scheme that correctly preserves the relevant slow degrees of freedom, and we test this idea for three systems of increasing complexity. We show that in contrast to this method existing coarse-graining schemes such as those from information theory or structure-based approaches are not able to recapitulate the slow time scales of the system.

## Abstract 2

Coarse-graining is a popular approach to study large and complex macromolecular systems. We propose the optimization of coarse-grain mapping to preserve the slow dynamical processes of a system.

## Introduction

Numerical simulation of complex high-dimensional systems in biophysics and condensed matter has become a powerful tool for the understanding of processes that can not be directly observed in wet lab experiments. The significant advances in hardware and software of the last couple of decades now allow one to routinely simulate medium size proteins at atomistic resolution and microsecond time scales. With dedicated hardware,^1^ or bias-enhanced sampling techniques,^2,3^ or distributed simulations combined with Markov state models (MSMs),^4^ it is possible to reach the millisecond time scale and sample folding and binding events and large conformational changes.^5−7^ From these long-time scale simulations, it is clear that the relevant structural, thermodynamic, and kinetic information for many biomolecular processes can be significantly simplified and expressed in lower-resolution models.^8−11^ Rare-event transitions such as folding, binding, and conformational changes can often be well described in terms of a few collective variables, as supported both by statistical mechanics arguments^12^ as well as plenty of empirical evidence resulting from transfer operator theory^8,13^ and Markov modeling.^4,14^ Consequently, it should be possible to summarize the essential properties of structure, thermodynamics, and kinetics that are relevant for the long-time scale behavior with a molecular model with fewer degrees of freedom.

Indeed, coarse-grained (CG) models, which implement this idea explicitly by representing groups of atoms as coarse-grained beads have long been used in the study of large molecular systems^10,15−17^ and have been useful to extend the reach of simulations to longer time scales and larger system sizes. The ability of a CG model to reproduce the relevant physics of a molecular system relies on two closely connected aspects: (1) the choice of the CG resolution and the corresponding degrees of freedom (usually referred to as “CG mapping”) and (2) the design and parametrization of the associated effective energy function. Several design principles to tackle the second of these tasks have been proposed to obtain a CG energy once the resolution is set. These include (i) bottom-up CG approaches via force-matching, relative entropy minimization, or Boltzmann inversion^18−20^ where the CG model is designed to reproduce the same coarse-grained thermodynamics of an all-atom model, (ii) fitting a set of observable quantities to the corresponding ones obtained in all-atom simulation and/or experimental data,^21,22^ or (iii) the minimization of frustration in model protein systems.^16^ In addition, the flexible parametrization of the CG energy using neural networks has recently received great attention.^23−29^

On the other hand, the systematic selection of a suitable CG mapping, i.e., which degrees of freedom to retain upon coarse-graining, is a task that has received comparatively little attention^30,31^ and is often left to the scientist’s chemical intuition. The success of a CG model is often assessed *a posteriori* by comparing the results of CG simulations with their all-atom counterpart or experimental data, however, such a comparison can not disentangle the effects associated with the CG mapping from the ones associated with the choice of energy function.^23,32−37^

One of the first approaches to quantify the “goodness” of a given CG mapping for the parametrization of a CG energy was introduced via the definition of the mapping entropy (*S*_map_) as part of a relative entropy framework^19^—also known as likelihood-based training of energy-based models in machine learning.^38^*S*_map_ depends only on the CG mapping (i.e., it is not affected by the choice of the CG energy), and its absolute value quantifies the amount of information lost upon coarse-graining. Following this idea, the minimization of the absolute value of *S*_map_ (or, equivalently, the maximization of its signed value, see Methods for details) has been proposed as a criterion for selecting a CG mapping at a given resolution.^39^ While the initial work was demonstrated on harmonic systems where *S*_map_ can be analytically computed, the applicability of the method has been later extended^40^ by deriving a numerical approximation of *S*_map_ that enables its estimation for more complex systems.^41,42^

In later work, it was noted that the maximization of *S*_map_ for the selection of an optimal CG mapping preserves upon coarse-graining mostly information associated with local high-frequency motions rather than global processes.^43^ The same authors proposed as an alternative approach the selection of a mapping scheme by optimizing a different quantity, the Vibrational Power, defined as the trace of the mass-weighted covariance matrix of the CG coordinates. This quantity allows an estimate of how well a CG model preserves large-scale motions.^43^

In parallel, different groups have proposed to define CG mapping schemes based on their ability to recover all-atom coordinates, e.g., by learning a CG mapping and all-atom reconstruction simultaneously via an autoencoder^25^ or by other machine learning approaches that employ structural classification or reconstruction errors (RE).^44−46^

In this work, we systematically investigate the effect of CG mapping on its ability to reproduce the long time scale processes of the system. We follow an orthogonal direction compared to previous approaches and exploit the Variational Approach for Markov Processes (VAMP)^47^ in order to propose the selection of an optimal CG mapping that explicitly maximizes the CG model’s ability to reproduce long-time scale processes. By means of a careful comparison, we find that such a VAMP-optimized CG mapping substantially disagrees with existing approaches.

## Results and Discussion

**Figure 1:**
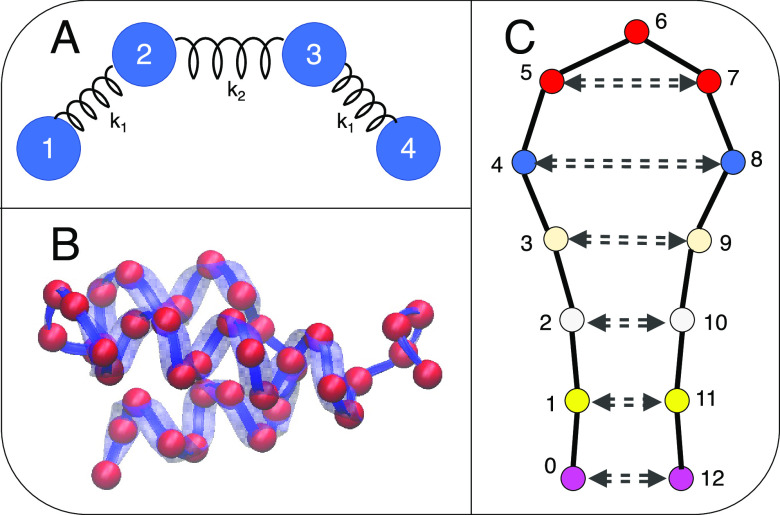
Visualization of the 3 systems studied. (A) Simple 4-bead harmonic chain. Here each bead is treated the same, and *k*_1_ and *k*_2_ represent the strength of the spring. (B) Gaussian network model from the C_α_ atoms of protein 2ERL with a neighbor cutoff of 10 Å. (C) Model protein system, as defined in ref (48), representative of a protein hairpin. Pairs of beads with equal color have attractive interactions.

We first discuss the general idea of our approach and then demonstrate it on three separate systems of increasing complexity (see Figure 1): (1) a 4-bead harmonic chain, (2) a Gaussian Network Model (GNM) of a protein, and (3) a model protein previously studied in the literature that is capable of adopting folded, misfolded, and unfolded conformations.^48^ For each choice of linear mapping  from the fine-grained coordinates  to the CG coordinates , we consider the effective CG energy, *W*(**X**), that is thermodynamically consistent with the full resolution model with energy *u*(**x**):^18^
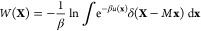
1where β is the inverse temperature. For systems 1 and 2, we can obtain the thermodynamically consistent CG energy function analytically, as well as the partition function and all ensemble averages (see the Supporting Information for detail). For system 3, the thermodynamically consistent CG energy can not be computed analytically, and one would need to use, e.g., a machine-learning approach to approximate it numerically.^23,26^ However, we do not need an expression for the CG energy to evaluate the different metrics for the selection of the optimal mappings as they can be computed from equilibrium trajectories of the underlying high resolution reference model (see the Supporting Information for detail).

We choose here to focus on simple and small systems instead of a more realistic protein model for ease of interpretation (system 1), to be able to obtain analytical results (system 2), and/or to enumerate all possible mapping choices and exhaustively compare the different metrics (system 3).

We show that in all cases a mapping scheme can be clearly and efficiently selected to best capture the long-time dynamics of a system even for highly nonlinear systems, a direct advantage over methods that must make linear approximations or ignore the time evolution dimension of the system.

### Defining the CG Mapping Criterion via the Variational Approach for Markov Processes (VAMP)

In the analysis of molecular dynamic (MD) simulations, one often seeks to define reaction coordinates that are able to characterize the slowest processes, or rare-event dynamics.^49^ On long time scales, the equilibrium molecular dynamics of molecules with rare events can be expressed in terms of the dominant eigenvalues and eigenfunctions of the dynamical propagator, such that these eigenfunctions are a natural choice for the rare-event coordinates.^4,13^ The Variational Approach for Conformation dynamics (VAC)^50,51^ is a framework to systematically approximate the rare-event eigenfunctions, which is achieved by maximizing the VAC score. When representing these functions with a linear combination of basis functions, one obtains the Time-lagged Independent Component Analysis (TICA) algorithm^52,53^ as a result. However, the score can also been used in a more general setting, for example, in the training of neural networks to find a nonlinear representation of the rare event coordinates.^54−56^ Recently, VAC has been generalized to the variational approach for Markov processes, which also permits the dynamics to be out of equilibrium^47^ and is closely connected to Koopman theory for dynamical systems.^57−59^

The VAMP score is a quantity that can be easily computed from simulated trajectories and assesses the ability of a set of variables to describe the slow dynamics of the system: the higher the VAMP score, the more appropriate the set of coordinates to serve this purpose. The VAMP score for reversible dynamics can be written as^47^
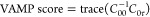
2
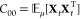
3
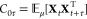
4where **X**_*t*_ is the vector containing the values of all the selected coordinates at time *t*, **X**_t+τ_ contains the values of such coordinates after a lag time τ, and  is the expectation value computed with the equilibrium probability distribution of the full resolution model. The matrices *C*_00_ and *C*_0τ_ are the covariance matrix and the time-lagged covariance matrix, respectively. For the harmonic systems studied here, the VAMP score, as well as other scores to assess the quality of the CG mapping can be computed analytically, except for the reconstruction error (See Methods for the derivation of these expressions).

Recently, the VAMP score has been used for selecting optimal features for constructing Markov state models such that the rare-event dynamics of the molecule are best-resolved.^60^ Here we propose to use the VAMP score in order to define the CG mapping: for a fixed resolution, the selection of CG degrees of freedom that best capture the long time scale dynamics also need to maximize the VAMP score among all possible CG mapping schemes.

In the following, we discuss the results on three model systems, while the detailed calculations are reported in the Methods section and in the Supporting Information.

### 4-Bead Model System

We start by examining a simple symmetric 4-bead harmonic chain (Figure 1A) with the Hamiltonian
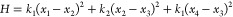
5For this system, we can obtain analytical results of thermodynamic and kinetic quantities and exhaustively enumerate all possible CG mapping schemes.

In the coarse-graining community, there are two primary styles of mappings: (i) “slicing”, whereby individual atoms are selected to represent the coarse-grained beads, or (ii) “averaging”, where multiple atoms are used to represent a bead and their properties averaged.^61^ Here we consider all possible slicing and averaging mappings from the 4 “atom” system into 2 CG beads. In the averaging mappings, we assign the same weight to each atom assigned to the same bead.

**Figure 2:**
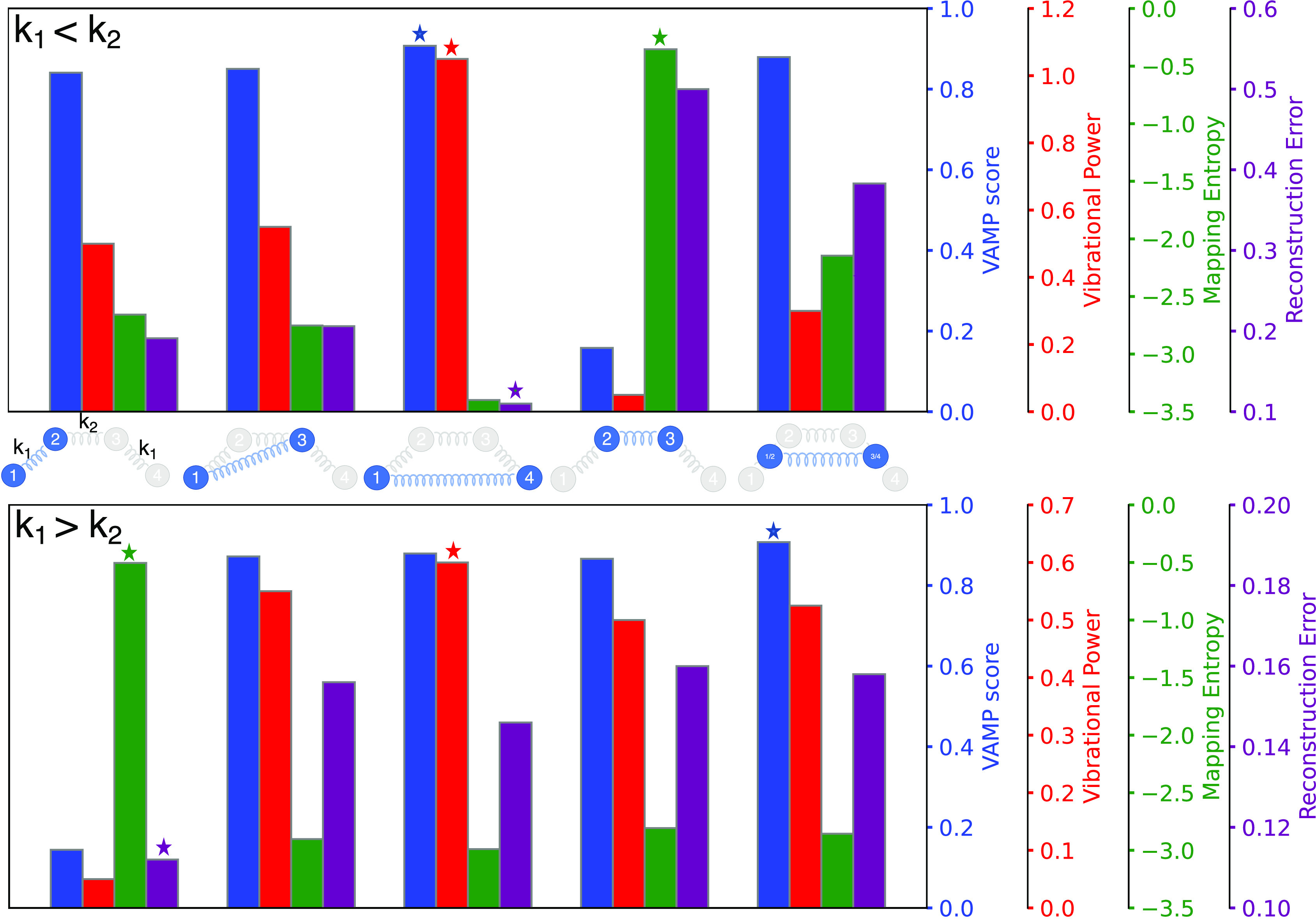
Comparison between different CG mapping methods for the 4-bead harmonic chain. The top part shows the results for the case *k*_1_ < *k*_2_ (soft spring at the edges) while the bottom part for *k*_1_ > *k*_2_ (soft spring in the center). The different mapping metrics are reported for each of the considered CG maps (illustrated by the cartoons in the middle), with the optimal values indicated by a star.

By varying the relative stiffness of the springs between the beads (*k*_1_ < *k*_2_ or *k*_1_ > *k*_2_) we obtain different optimal mappings (Figure 2). In the case of a stiffer spring in the middle and softer springs at the edges (*k*_1_ < *k*_2_), a CG mapping retaining beads 2 and 3 only describes the fast fluctuations that are usually not of interest. However, this is the CG mapping scheme maximizing the (negative) value of the mapping entropy. On the other hand, the maximization of the Vibrational Power and of the VAMP score, as well as the minimization of the reconstruction error, yields the same CG mapping corresponding to the selection of the first and last bead of the chain, therefore describing the largest (and, in this case, slowest) fluctuations.

The situation is quite different for the case of a softer spring in the middle (*k*_1_ > *k*_2_), which can be considered a toy model of a molecule where hydrogen atoms are bound with a very stiff spring to two central atoms (e.g., hydrogen peroxide H–O–O–H). As high energy fluctuations have a dominant effect on the evaluation of *S*_map_, the maximization of the (negative) value of *S*_map_, in this case, selects to preserve the fast motion associated with the first two (or equivalently the last two) beads. Interestingly, this is the mapping scheme also selected by the minimization of the reconstruction error. The maximization of the Vibrational Power again preserves the motion of the largest amplitude,selecting the first and last beads. However, in this case, this CG mapping does not correspond to the one preserving the slowest motion, which is instead given by the maximization of the VAMP score and yields an averaging scheme that takes into account also the contribution of the two middle beads (see Figure 2).

The physical meaning of the difference between the maximization of the Variational Power and the maximization of the VAMP score is analogous to the difference in the selection of reaction coordinates by methods such as Principal Component Analysis (PCA), which describes the *largest amplitude* motions, as opposed to methods such a TICA, describing the *slowest* processes. It is well-known,^53^ that PCA and TICA can give very different results as the amplitude of a motion does not necessarily report on its time scale. Therefore, if the interest is the preservation of the slowest processes upon coarse-graining, once the resolution is selected, the CG mapping maximizing the VAMP score should be considered.

### Gaussian Network System

In order to further illustrate the divide between the different mapping criteria but still be able to obtain analytical solutions, we consider a Gaussian Network Model (GNM) of a protein. Gaussian network models have been used extensively in the past for proteins as their simplicity allows for fast, interpretable results that in general give qualitative agreement with experimental results.^62,63^ GNMs typically select carbon alpha (C_α_) atoms in the protein backbone and model with a harmonic spring the interaction between any C_α_ pairs that lie within a preset cutoff distance in the native structure.^64^ As usual for GNMs, all these springs are modeled with equal spring constants, so in contrast to the 4-bead system above, the GNM dynamics depend on the degree of connectivity between beads rather than the strength of individual springs.

Following ref (43), we consider the GNM of the 40-residue protein 2ERL. Given the small size of the protein, we can enumerate a large number of CG mappings for different numbers of CG beads. We consider all the possible partitions of the 40 C_α_’s into *N* = 2, 3, 4, 5, and 6 groups of subsequent atoms, and define the CG beads as the average over each group of atoms. It is worth noting that our definition of possible mappings is different from what is considered in ref (43). There, the authors define a valid mapping as a partitioning of all the 40 C_α_’s of protein 2ERL into *N* = 2, 4, 5, 8, 10, 20 disjoint groups of an equal number of 40/*N* C_α_ atoms. They require the C_α_ atoms belonging to the same CG bead to be connected in the GNM but not necessarily to be subsequent along the sequence. This criterion generates quite a large number of possible mappings, e.g., for *N* = 5 there exist ∼10^24^ choices. For this reason, they sample the landscape of allowed CG mappings with Monte Carlo simulations. In contrast to ref (43), in the present work we require the CG beads to be formed by groups of subsequent atoms, while we also consider partitions of atoms into groups of different sizes, ranging from 1 to 40–(*N*–1). This definition of possible mappings allows us to exhaustively enumerate them. For instance, for *N* = 5 there exist 82251 combinations. A more detailed comparison of the results obtained with the mapping choice of ref (43) is presented in the Supporting Information.

**Figure 3:**
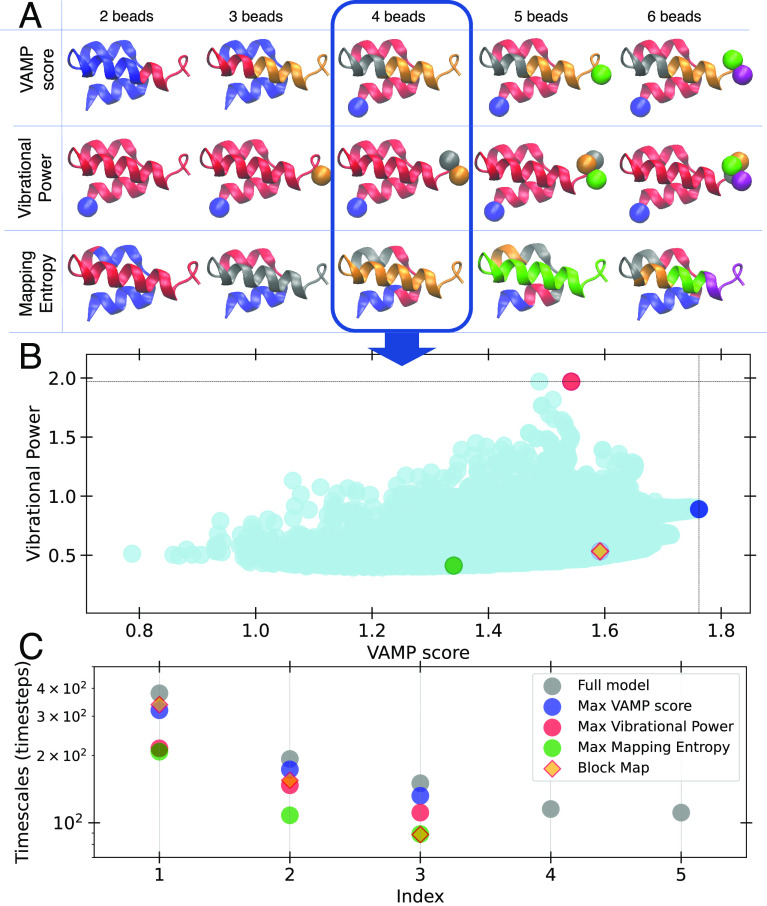
(A) Comparison of the optimal mappings for the GNM of protein 2ERL at different bead resolutions. The CG mappings optimizing the VAMP/Variational Power/mapping entropy (top/middle/bottom) criteria (refer to text for details). Different resolutions are shown from the left (2 CG beads) to the right (6 CG beads). (B) VAMP score corresponding to each possible mapping strategy at 4-bead resolution plotted versus the corresponding Vibrational Power. The gray lines running along the top and right indicate the optimal mappings for the two strategies. The colored dots indicate the models corresponding to different optimal mapping strategies, and the orange diamond correspond to the block map, with all beads composed of the same number of atoms. (C) Time scales of the system (as obtained by TICA) ordered by decreasing value for the three different mapping strategies (indicated by the colors) for a model with a 4-bead resolution, in comparison to the full-resolution model.

At each resolution, we evaluate the analytical expression for the VAMP score, the Vibrational Power, and *S*_map_ (see Methods and Supporting Information for details) over all the possible CG mappings and select the ones optimizing the different metrics. Because of the large number of possible CG mappings for this system, the training of an autoencoder for a numerical estimate of the reconstruction error for each of them is not feasible and we limit the analysis to these three metrics that can be analytically evaluated. Figure 3A shows the grouping of consecutive C_α_ atoms to CG beads by different colors. Groups consisting of single C_α_’s are shown as a sphere. It is clear that different mapping criteria lead to significantly different optimal mapping schemes. Analogously to the 4-bead harmonic system discussed above, the maximization of the Vibrational Power tends to select *N* – 1 single C_α_ atoms at the termini as CG beads while grouping all the remaining 40 – (*N* – 1) C_α_ atoms into a single bead. This effect is seen at all resolutions considered (see Figure 3A). Indeed, this selection is consistent with the preservation of the largest amplitude of motion, corresponding to the displacements of the termini of the protein with respect to each other. On the other hand, the optimization of the *S*_map_ yields CG mappings corresponding to more uniform partitions of atoms along the sequence, consistent with the preservation of local and high-frequency motions. The CG mappings selected by the maximization of the VAMP score is a sort of compromise between the grouping selected according to the optimization of the *S*_map_ or Vibrational Power metrics, as it comprises a combination of single atoms at the termini and different stretches of atoms across the protein.

We note the optimal mappings for the *S*_map_ or Vibrational Power metrics shown in Figure 3 are very different from those reported in ref,^43^ as there the CG beads are constrained to contain the same number of C_α_ atoms and the nonhomogeneous bead sizes obtained here are *a priori* excluded.

In Figure 3B, the VAMP score corresponding to all possible 4 bead resolution CG mappings is plotted versus the Vibrational Power, and the CG optimal mappings according to different criteria are highlighted.

Figure 3C shows that VAMP-based CG maps best preserve the long time scale dynamics. The time scales are estimated from the eigenvalues of the Koopman matrix defined by the covariance and time-lagged covariance of the coordinates of the CG beads, as customary in the analysis of MD simulation (see Supporting Information and ref (53) for additional detail). The time scales are reported in order of decreasing value for the different optimal mapping choices at resolution *N* = 4 and compared with the time scales estimated by the same method on the full resolution (i.e., *N* = 40). Based on the definition of the GNM energy function (eq 6), if a resolution of 4 beads is chosen for the CG system, we can compare only the three slowest time scales of the full resolution GNM with the time scales reproduced by the different CG systems. The CG mapping selected by the optimization of the VAMP score reproduces accurately the first three time scales, significantly better than the ones selected by the optimization of the Vibrational Power or of *S*_map_. The orange diamonds in Figure 3, parts B and C, mark the values corresponding to the block map, that is the CG map where each bead contains the same number of atoms. This map is very close to the one optimizing the Vibrational Power when the mapping space is defined by the criterion of ref (43) (see Supporting Information for a detailed comparison with the results obtained with this criterion).

### Model Protein System

Finally, we turn to a more realistic albeit simple system: a 13-atom model protein containing harmonic bonds/angles and nonbonded interactions via Lennard-Jones potentials (Figure 1C). This system was originally proposed in ref,^48^ and shown to exhibit folding/unfolding dynamics. For increased interpretability and for fast computation, we focus here only on the slicing strategy for the definition of the CG mappings, and at the fixed resolution of *N* = 5 CG beads, considering all  ways of selecting 5 beads from the 13 “atoms” of the model. Because of the nontrivial interactions, we cannot obtain analytical resolutions for this system, however, the VAMP score and Vibrational Power can be computed numerically by estimating the covariance and time-lagged covariance matrices over simulated trajectories^47^ (see Supporting Information). For every mapping, we assume that the corresponding effective CG energy function is thermodynamically consistent with the reference fine-grained model. This implies that the CG model can reproduce the same probability distribution for the CG degrees of freedom as obtained from the high-resolution trajectories under the CG mapping. With these premises, we can use the high-resolution trajectory directly to compute ensemble averages.

The relatively small number of CG mapping choices allows training a separate autoencoder for each mapping in order to evaluate the corresponding reconstruction error, whereas values of *S*_map_ cannot be as simply estimated and associated with the different mappings for this system. A numerical approximation for *S*_map_ does exist;^40^ however, some ambiguity remains on the definition of the appropriate choice of parameters for this method. We found the results to vary wildly with small variations of these parameters. Additionally, as it was already discussed in ref (43), and as is evident from the results presented above, it is clear that *S*_map_ does not provide useful information on the preservation of slow degrees of freedom upon coarse-graining.

**Figure 4:**
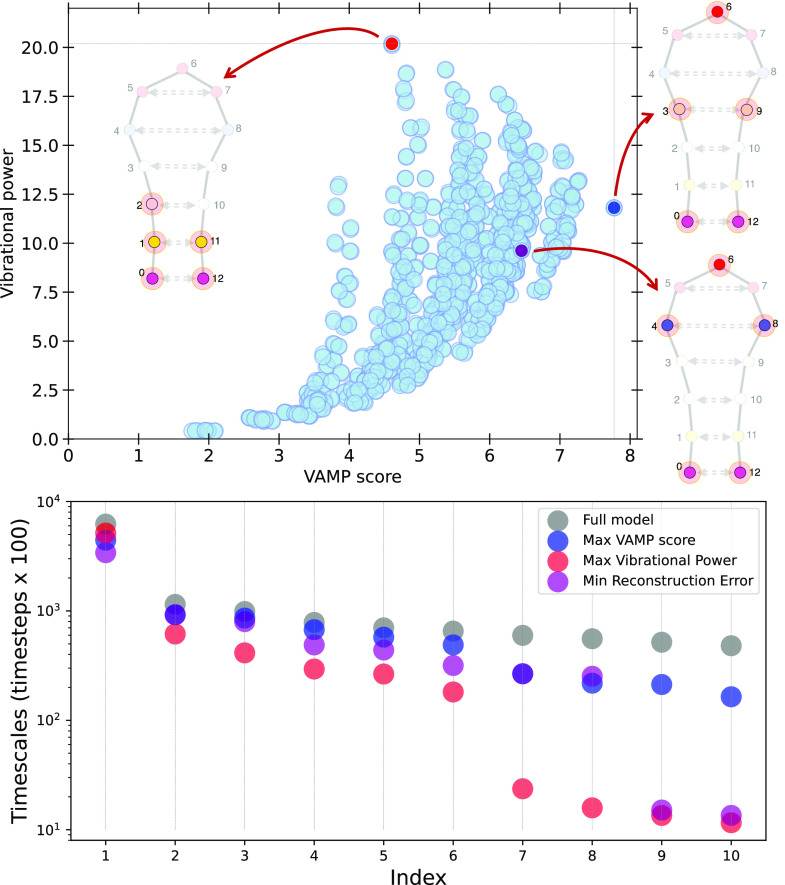
Top panel: The VAMP score corresponding to all possible 5 bead resolutions CG mappings for the model protein is plotted versus the Vibrational Power. The gray lines running along the top and right indicate the optimal mappings for the two strategies. For each optimal mapping, the corresponding CG model is shown with the selected beads highlighted. Bottom panel: Projection of the TICA time scales for CG models corresponding to different optimal mapping strategies (colored dots), compared to the TICA time scales of the full-resolution model (gray dots).

Figure 4a shows the Vibrational Power corresponding to each of the different CG mappings, plotted as a function of the VAMP score for the same mapping. The two quantities weakly correlate in the area corresponding to poor choices of CG mapping, indicated by small values of both. However, the CG mappings selected by the optimization of these two metrics (gray horizontal and vertical lines for the Vibrational Power and VAMP score, respectively, and illustrated on a cartoon of the protein model) are significantly different: a maximum Vibrational Power select the atoms at the terminal ends as CG beads, while a maximum VAMP score disperses them evenly throughout the model protein. As for the previous systems, these results are in agreement with the interpretation of the VP metric capturing the largest amplitude displacements of the system, but failing to recover the more nuanced motion involving degrees of freedom along the hairpin. The CG mapping corresponding to a minimal reconstruction error is also highlighted in the plot and illustrated on the protein model.

As in the previous example, Figure 4b shows the model protein’s relaxation time scales recovered by different CG mappings. The time scales are approximated using the TICA method,^65^ by computing the Koopman matrix using covariance matrices of all interparticle distances (see Supporting Information for details). The 6 slowest time scales associated with the CG mapping with optimal VAMP score (4b, blue dots) closely match the time scales of the 6 slowest time scales of the full resolution model (gray dots). Additionally, even faster time scales (index >6) are reproduced to a good degree. The CG mapping with maximum Vibrational Power reproduces the 6 slowest time scales to a lesser extent, and presents a sharp drop for time scales with an index >6, again indicating that this metric does not necessarily preserve the system dynamics. This gives evidence that, for this system coarse-grained at a 5-bead resolution, the optimization of the Vibrational Power captures well the longest-time scale behavior of the system corresponding to the fluctuations of the end-to-end distance, but it cannot recover additional slow processes. The results associated with the CG mapping corresponding to the minimization of the reconstruction error appear to lay in between what is obtained with the other two metrics.

It is important to note that, if different or additional properties are desired in a CG model (instead or in addition to recovery of the slow dynamics), e.g., the possibility of accurately backmapping to the full resolution, one needs to change the optimization metric or find a compromise between two or more optimization criteria. While the maximization of the mapping entropy appears to produce mappings very different from the optimization of the other metrics in all the examples presented here (see, e.g., Figure 2), from Figure 4, it appears that there is no strong trade-off between the minimization of the reconstruction error and the maximization of the VAMP score, at least for this simple model system.

## Conclusion

We explore the definition of an optimal CG mapping scheme and consider a variety of methods based on information theory, structural reconstruction, or Koopman theory for dynamical systems. We do this under the assumption that coarse-graining can be split into two separate processes: (1) choice of mapping and (2) definition of a CG Hamiltonian. Here we focus on the choice of mapping to build CG models with a well-defined main objective and use the thermodynamic consistency criterion for bottom-up coarse-graining^18^ to define the corresponding CG Hamiltonian (eq 1). While this assumption is justified for the simple models used in this work, in a more realistic setting the choice of the mapping is of course interconnected with the design of the CG energy function. In biological and soft matter systems, we argue that the main objective of coarse-graining is to be able to correctly capture the behavior of a complex and high-dimensional system over long time scales. That is, we want a coarse-grained model to simulate the time scales where most physically relevant processes such as global protein conformational changes or ligand binding/unbinding occur, but they are challenging to characterize with fine-grained simulations. The faster degrees of freedom in contrast, which may be potentially relevant for, e.g., biochemical specificity, are amenable to be probed with all-atom simulations at a reasonable computational cost. Following this line of reasoning, a suitable bottom-up coarse-grained model should be able to accurately recover the appropriate slow mechanisms and describe the transitions between the same metastable states as accurately as the all-atom counterpart. As the preservation of the slowest processes upon coarse-graining is of key importance, a mapping scheme optimized toward this goal is usually desirable.

In the field of dynamical systems and Koopman theory, the VAMP score has been introduced to quantify the ability of a (small) set of features to capture the slow “dynamical modes” of a system, and we propose here to use this metric also for the definition of an optimal CG mapping. Loosely speaking, a choice of coordinates based on the maximization of the VAMP score leads to the selection of the subset of degrees of freedom that more accurately spans the space defined by the first few eigenfunctions of the Koopman operator, that in turn provides the best linear approximation of the system’s dynamic evolution.^47^

It is important to note that, for realistic systems, in order to accurately recover the dynamics of the fine-grained system in the CG coordinates, one would need to use a generalized Langevin equation, derived, e.g., through the Mori–Zwanzig formalism and comprising a memory kernel. However, building up on previous work,^66,67^ we have recently shown^68^ that for overdamped Langevin dynamics, if the eigenfunctions of the Koopman operator of the fine-grained system can be well approximated in the space spanned by the CG coordinates, the corresponding time scales are also well approximated by the projected CG dynamics in the form of an overdamped Langevin equation. As the maximization of the VAMP score selects the CG coordinates that best approximate the fine-grained eigenfunctions, the maximization of the VAMP score is consistent with this point of view.

We have tested this idea by comparing the performance of a CG mapping scheme maximizing the VAMP score against other popular choices, on three different systems of increasing complexity, by means of both analytical and numerical calculations. We show that, while the optimization of the VAMP score leads to the successful recovery of the dynamics on the slowest time scales, alternative methods fall short in this regard.

On the basis of these results, we believe that the definition of CG mapping to preserve molecular kinetics can be done systematically. Here we have shown a proof of principle on simple model systems, but the same criterion could be used for the choice of resolution in a more realistic CG protein model transferable in sequence space. That means that different partitioning of the atoms in each amino acid into CG beads could be explored and compared. For instance how much more kinetic information is preserved if a C_α_–C_β_ CG model is used instead of a C_α_-only model on a set of test proteins? Future work will address this question and related ones.

In this regard, we want to emphasize that the choice of mapping is only the first step in constructing a complete CG model and the overall performance of the model critically depends on the definition of the CG Hamiltonian as well. Nevertheless, we believe that the optimization of the CG resolution plays an important role in the ability of the model to reproduce the system’s long-time scale behavior.

## Methods

### Simulation Protocol

Numerical simulations for the harmonic systems were carried out following the protocol outlined in ref (69) and are summarized in the Supporting Information.

### Coarse-Graining of a Harmonic Model

We consider a general harmonic system with an energy function in the form
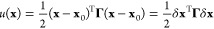
6where **Γ** is the connectivity matrix (called Kirchhoff matrix in the context of a GNM), **x** is the vector of all coordinates, **x**_0_ is the vector of coordinates of a reference configuration, and δ**x** is the displacement vector.

By following ref (39), the full thermodynamics of a harmonic system can be obtained, as well as for CG models thermodynamically consistent with it. Here we report the definitions and final analytical expressions; for a full derivation please refer to refs (39 and 43).

We assume a linear mapping  defines the coarse-grained coordinates  from the fine-grained ones : **X** = *M***x**, with *N* ≪ *n*. eq 1 reports the general definition of the effective CG energy function, *W*(**X**), that is thermodynamically consistent with a fine-grained model with energy *u*(**x**).^18^ For a harmonic system with energy given by eq 6, a compact solution for the expression of the thermodynamically consistent CG energy (eq 1) can be obtained analytically:
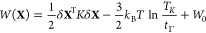
7where *W*_0_ is a protein-independent constant, δ**X** = **X** – **X**_0_ is the vector of the displacements in the coordinates of the CG beads, **X**_0_ = *M***x**_0_ is the reference configuration of the CG model, and we have defined the effective CG matrix *K* as
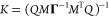
8where *Q* = **1**_*N*_ – 1/*N***J**_*N*_**J**_*N*_^*T*^ is the projection operator that filters out free translations and the vector . In eq 7
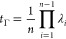
9and
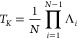
10are the products of the nonzero eigenvalues of the matrix **Γ** and *K*, respectively.

### Mapping Entropy

Following ref (39), the mapping entropy associated with a CG model is given by the difference between the excess configurational entropy of the full resolution model, *s*_*r*_, and the same quantity when it is “perceived” from the CG configurational space, *s*_*R*_:
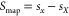
11
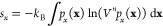
12
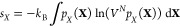
13where *V* is the volume of the system, and *p*_*x*_(**x**) and *p*_*X*_(**X**) are the Boltzmann weights associated with the energy function of eq 6 and of eq 7, respectively. The mapping entropy *S*_map_ is always negative and its maximization (that is, the minimization of its absolute value) has been proposed as a criterion to define an optimal CG map.^39^

For a harmonic system, these expressions can be analytically evaluated,^39,43^ and they give
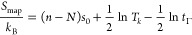
14
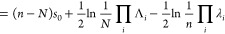
15
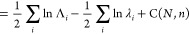
16where both *s*_0_ and C(*N*, *n*) are model-independent constants (*s*_0_ is only function of the volume, and C(*N*, *n*) depends on the dimensionality of the fine-grained and coarse-grained models), and *t*_Γ_, *T*_*K*_ are the products of the nonzero eigenvalues, λ_*i*_ and Λ_*i*_ of the matrices Γ and *K*, respectively (see eqs 9 and 10)).

As the eigenvalues λ_*i*_ and Λ_*i*_ are all positive, the expression for the mapping entropy (16) can also be written as
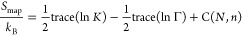
17
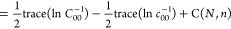
18
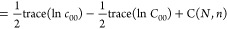
19where we have used *C*_00_ = *K*^–1^ and defining *c*_00_ = Γ^–1^.

### VAMP Score for Harmonic Systems

Here we provide a quick overview of the procedure and provide the final result for the analytical calculation of the VAMP score in a system of beads connected by harmonic springs, with energy in the form of eq 6. For the full derivation please refer to the Supporting Information.

From its definition (eq 2), the calculation of the VAMP score requires the evaluation of the matrices *C*_00_ and *C*_0τ_ (eqs 3 and 4). These matrices can be computed analytically for a harmonic system. The matrix *C*_00_ represents the covariance of the CG coordinates and is straightforwardly obtained as
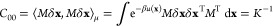
20where *K* is the effective CG matrix defined in eq 8. The matrix *C*_0τ_ is the time-lagged covariance matrix, that can be expressed as
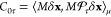
21where  is the *propagator* of the dynamics associated with the (full resolution) harmonic system, for a lagtime τ, and  is the *generator* of the dynamics.^13^ Assuming that the time evolution of the system can be described as an overdamped Langevin dynamics, with friction coefficient γ, the eingenfunctions and eigenvectors of the dynamic propagator can be obtained. Therefore, by decomposing the system coordinates into these eigenfunctions, expression 21 can be analytically evaluated (see Supporting Information for details). The final result is
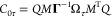
22where the matrix Ω_τ_ is defined as
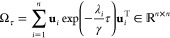
23where λ_*i*_ and **u**_*i*_ are the eigenvalues and eigenvectors of the matrix **Γ**. With the expressions for *C*_00_ and *C*_0τ_, the VAMP score is obtained as
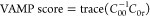
24
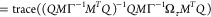
25

### Vibrational Power

Following ref (43), the vibrational power is defined as the trace of the mass-weighted covariance matrix describing correlated fluctuations. Here we assume uniform mass for all particles, and therefore, in our notation, the vibrational power (VP) of a CG model defined by the CG mapping *M* is
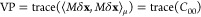
26

### Reconstruction Error

The reconstruction error (RE) is defined as the mean square error (MSE) between the original coordinates of a fine-grained configuration and the reconstructed fine-grained coordinates of the corresponding CG configuration:
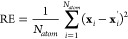
27Here **x**_*i*_ indicates the original fine-grained coordinates of atom *i* and **x**_*i*_^′^ the reconstructed fine-grained coordinates. As detailed in the Supporting Information, the reconstruction of the fine-grained coordinates from a CG configuration is obtained by means of an autoencoder, where the encoder part is defined by the CG mapping and the decoder part is trained on long equilibrium simulations of the fine-grained model.

## Data Availability

Simulation data and the code to reproduce the analysis and the plots shown in the manuscript are accessible at https://github.com/ClementiGroup/cg-mapping.git.

## Supporting Information Available

Details on numerical simulations and data analysis and on the definition and numerical calculation of the reconstruction error, additional details on the analytical and numerical calculations of the VAMP score, and detailed comparison with the results of ref (43) (PDF)
