# Supplementary material for: Slicing and Dicing: Optimal Coarse-Grained Representation to Preserve Molecular Kinetics
Source: ACS Cent Sci. 2023 Jan 17;9(2):186–96. doi: 10.1021/acscentsci.2c01200 (PMC9951291; doi:10.1021/acscentsci.2c01200)
Supplement: Supplementary file 1 — oc2c01200_si_001.pdf [file oc2c01200_si_001.pdf]

Slicing and Dicing: Optimal Coarse-grained Representation to Preserve Molecular Kinetics

Wangfei Yang,[†,‡] Clark Templeton,[¶] David Rosenberger,[¶] Andreas Bittracher,[§] Feliks Nüske,[‖] Frank Noé,[§,¶,†,⊥] and Cecilia Clementi[*,¶,†,#,⊥]

†*Center for Theoretical Biological Physics, Rice University, Houston, TX 77005, USA.*

‡*Graduate Program in Systems, Synthetic and Physical Biology, Rice University, Houston, TX 77005, USA.*

¶*Department of Physics, Freie Universität Berlin, Arnimallee 12, 14195 Berlin, Germany.*

§*Department of Mathematics and Computer Science, Freie Universität Berlin, Arnimallee 12, 14195 Berlin, Germany.*

‖*Max Planck Institute for Dynamics of Complex Technical Systems, Sandtorstr. 1, 39106 Magdeburg, Germany.*

⊥*Department of Chemistry, Rice University, Houston, TX 77005, USA.*

#*Department of Physics, Rice University, Houston, TX 77005, USA.*

E-mail: cecilia.clementi@fu-berlin.de

Numerical simulations

Numerical simulations for the harmonic systems were carried out with an effective temperature of $k_B T = 1$ energy unit, $\gamma = 0.1$ inverse time, dt $= 0.001$ time unit, $k = 1$ for the GNM of protein 2ERL, and $k_1, k_2 = (1, 10)$ for the 4-bead harmonic system. Langevin integration could be implemented directly in the jupyter notebook. Please refer to the associated GitHub repo for the codes.

The dynamics of the model protein were simulated by using the simulation parameters outlined in[1] with the only modification that k_BT=1.33 instead of $k_BT = 1$. A single long trajectory of 50 million timesteps was generated, saving the coordinates every 5 steps. All simulations were carried out in OpenMM.[2]

Simulation Analysis

Here we show (see Fig. S1) the implied timescale plot for the model protein, as obtained by TICA,[3,4] as a function of the TICA lagtime, and the plot of the end-to-end distance as a function of time (see Fig. S2). The first is used to determine a suitable lagtime for the system, for the calculation of the VAMP score. The second is to justify the slightly higher temperature of the system compared to that from.[1] Refer to the Figures themselves for a more in-depth description.

Reconstruction Error

Autoencoder Architecture and Training Strategy

The reconstruction error (RE) is calculated by training an autoencoder model whose encoder part performs the coarse-grained (CG) mapping and the decoder part reconstructs the fine-grained (FG) structure using the CG coordinates as its input. We define the reconstruction error as the mean square error between the original coordinates and the reconstructed ones:

$$RE = \mathrm{mean}((\mathbf{x}_{rec} - \mathbf{x}_{FG})^2) \tag{1}$$

where $\mathbf{x}_{FG}$ are the FG coordinates and $\mathbf{x}_{rec}$ the reconstructed ones, and the average is performed over an ensemble of configurations (from long MD trajectories). In this work, for each considered CG mapping, the encoder part is linear and it's fixed by the mapping. Then,

with the CG coordinates as input, the decoder part is trained to minimize the reconstruction error over training data sampled from equilibrium simulations.

The decoder consists of two modules, a linear and a non-linear one as shown in Fig. S3. The linear module is a $3N \times 3n$ matrix transforming the $3N$ CG coordinates into $3n$ fine-grained ones, while the non-linear module is a neural network. The two modules are combined in a residual network architecture:

$$\mathbf{x}_{rec} = D_{linear}(\mathbf{x}_{CG}) + D_{non-linear}(D_{linear}(\mathbf{x}_{CG})) \quad (2)$$

where $\mathbf{x}_{CG}$ are the CG coordinates. We found that this design allows a faster convergence of the training than a decoder consisting only of a non-linear component. In practice, we first train the linear module till convergence, then add the non-linear correction and train it till convergence.

Data Set and Hyper-parameters

The hyper-parameters of the autoencoder, the data set and the training for the 4-bead and the model protein systems are shown in Table 1. The data used are obtained from MD simulation trajectories whose details have been described above. Cross-validation is applied in the training. In Fig. S4, we show the validation loss corresponding to the training of the CG mapping with the minimum RE, for the 4-bead, and model protein systems. The 2-stage convergence is caused by our separate two-step (linear and non-linear) training strategy.

Analytical VAMP score for harmonic systems

As discussed in the main text, the VAMP score is defined as

$$\text{VAMP score} = \text{trace}\left(C_{00}^{-1} C_{0\tau}\right) \quad (3)$$

Table 1: The hyper-parameters of the autoencoder, the data set, and the training.

Parameter	4-bead	Model Protein
Batch Size	4096	4096
Learning Rate (linear)	2e-4	1e-3
Learning Rate (non-linear)	2e-4	1e-3
Epoch (linear)	200	120
Epoch (non-linear)	200	230
Number of Hidden Layers	3	3
Width of Hidden Layers	$3n$	$3n$
Activation Function	Relu	Relu
Stride	10	100
Number of Frames	1000000	495000
Cross Valid Ratio	80%/20%	80%/20%

with the covariance matrix C_{00} and time-lagged covariance matrix $C_{0\tau}$ defined as:

$$C_{00} = \mathbb{E}_{\mu}\left[\delta\mathbf{X}_t \delta\mathbf{X}_t^{\top}\right] \tag{4}$$

$$C_{0\tau} = \mathbb{E}_{\mu}\left[\delta\mathbf{X}_t \delta\mathbf{X}_{t+\tau}^{\top}\right] \tag{5}$$

where $\mathbf{X}_t$ (or $\mathbf{X}_{t+\tau}$) is a set of features of the coarse-grained model evaluated at time t (or $t+\tau$ where τ is a lagtime), $\mathcal{P}_\tau = \exp(\mathcal{L}\tau)$ is the *propagator* of the dynamics associated with the (full resolution) harmonic system, for a lagtime τ, and $\mathcal{L}$ is the *generator* of the dynamics.[5] If we take the features to be the displacement of the Cartesian coordinates, the covariance matrix C_{00} is easily evaluated as

$$C_{00} = \langle M\delta\mathbf{x}, M\delta\mathbf{x}\rangle = K^{-1} \tag{6}$$

where $K = \left(QM\mathbf{\Gamma}^{-1}M^{\top}Q\right)^{-1}$ is the effective CG interaction matrix, and as in,[6] the matrix $Q = \mathbf{1}_N - \frac{1}{N}\mathbf{J}_N\mathbf{J}_N^{\top}$ is the projection operator that filters out free translations with the vector $\mathbf{J}_N = (1,\ldots,1)^{\top} \in \mathbb{R}^N$. In the previous expression the inverse $\mathbf{\Gamma}^{=1}$ is defined by removing

the zero eigenmode (that is, it's a pseudoinverse):

$$\mathbf{\Gamma}^{-1} = \sum_{i=1}^{n-1} \mathbf{u}_i \frac{1}{\lambda_i} \mathbf{u}_i^\top \tag{7}$$

where $\mathbf{u}_i$ are the normalized eigenvectors.

On the other hand, the evaluation of the time-lagged covariance matrix requires some additional calculations, as detailed below.

We assume an overdamped Langevin dynamics in a potential energy $u(\mathbf{x})$ for the time evolution of the system:

$$d\mathbf{x}_t = -\gamma^{-1} \nabla u\left(\mathbf{x}_t\right) dt + \sqrt{2 k_B T \gamma^{-1}} d\mathbf{B}_t \tag{8}$$

where γ is the friction coefficient and $\mathbf{B}_t$ is a Brownian motion.

The generator $\mathcal{L}$ associated with this dynamics is the differential operator acting on a generic function $f(\mathbf{x})$ as follows:

$$\mathcal{L} f(\mathbf{x}) = -\gamma^{-1} \nabla u \cdot \nabla f(\mathbf{x}) + \gamma^{-1} k_B T \Delta f(\mathbf{x}) \tag{9}$$

In the specific case of a harmonic system with potential energy $u(\mathbf{x}) = \frac{1}{2} \delta\mathbf{x}^\top \mathbf{\Gamma} \delta\mathbf{x}$, we have:

$$\mathcal{L} f(\mathbf{x}) = -\gamma^{-1} \mathbf{\Gamma}\mathbf{x} \cdot \nabla f(\mathbf{x}) + \gamma^{-1} k_B T \Delta f(\mathbf{x}) \tag{10}$$

With a change of variables from $\mathbf{x}$ to $\boldsymbol{\eta} = \mathbf{U}^\top \delta\mathbf{x}$, and using the eigenvalue decomposition of the interaction matrix $\mathbf{\Gamma} = \mathbf{U}\mathbf{\Lambda}\mathbf{U}^\top$, where $\mathbf{\Lambda}$ is the matrix of the eigenvectors of $\mathbf{\Gamma}$ the above equation becomes the sum of decoupled expressions along the normal modes:

$$\mathcal{L} f = -\gamma^{-1} \sum_{i=1}^{n} \left[\eta_i \lambda_i \frac{\partial}{\partial \eta_i} + k_B T \frac{\partial^2}{\partial \eta_i^2} \right] f \tag{11}$$

The (properly normalized) eigenfunctions $\psi_k(x)$ and eigenvalues ξ_k of the generator $\mathcal{L}$ (Eq.

10) for the 1-dimensional case (with potential energy $u(x) = \frac{1}{2}\lambda x^2$) are:[7]

$$\psi_k(x) = \frac{1}{k!}\left(\frac{k_BT}{\lambda}\right)\exp\left(-\frac{\lambda x^2}{2k_BT}\right)H_k\left(x\sqrt{\frac{\lambda}{2k_BT}}\right) \tag{12}$$

$$\xi_k = -\frac{\lambda}{\gamma}k, \quad \forall k = 0, 1, 2, \ldots \tag{13}$$

where $H_k(x)$ are the Hermite polynomials. The eigenfunctions are orthonormal with respect to the Boltzmann measure:

$$\int \psi_{k_1}(x)\psi_{k_2}(x)\exp\left(-\frac{\lambda x^2}{2k_BT}\right)dx = \delta_{k_1,k_2} \tag{14}$$

The analytical expression for the eigenfunctions of the generator $\mathcal{L}$ and propagator $\mathcal{P}_\tau$ of the multidimensional process (Eq. 11) is then:

$$\psi_{(k_1,k_2,\ldots,k_n)} = \tag{15}$$

$$\exp\left(-\frac{\sum_i \lambda_i\eta_i^2}{2k_BT}\right)\prod_{i=1}^{n}\frac{1}{k_i!}\left(\frac{k_BT}{\lambda_i}\right)^{\frac{k_i}{2}}H_{k_i}\left(\eta_i\sqrt{\frac{\lambda_i}{2k_BT}}\right) \tag{16}$$

with the indexes $k_i = 0, 1, \ldots\ \forall i \in [1, n]$. The corresponding eigenvalues of the propagator $\mathcal{P}_\tau$ are then:

$$\xi_{(k_1,k_2,\ldots,k_n)} = \exp\left(-\frac{\sum_i \lambda_i k_i}{\gamma}\tau\right) \tag{17}$$

By using the normal mode decomposition:

$$\delta\mathbf{x} = \mathbf{U}\boldsymbol{\eta} \tag{18}$$

and the orthonormality of the eigenfunctions (Eq. 14), the expansion of the coordinate

displacement $\delta\mathbf{x}$ in terms of these eigenfunctions reduces to a finite sum:

$$\delta\mathbf{x} = \tag{19}$$

$$= \sum_{k_1=0,1,\ldots} \sum_{k_2=0,1,\ldots} \cdots \sum_{k_n=0,1,\ldots} \langle \delta\mathbf{x}, \psi_{(k_1,k_2,\ldots,k_n)} \rangle \psi_{(k_1,k_2,\ldots,k_n)} = \tag{20}$$

$$= \langle \delta\mathbf{x}, \psi_{(1,0,\ldots,0)} \rangle \psi_{(1,0,\ldots,0)} + \langle \delta\mathbf{x}, \psi_{(0,1,\ldots,0)} \rangle \psi_{(0,1,\ldots,0)} + \tag{21}$$

$$+ \cdots + \langle \delta\mathbf{x}, \psi_{(0,0,\ldots,1)} \rangle \psi_{(0,0,\ldots,1)} \tag{22}$$

and the projections are easily evaluated as:

$$\langle \delta\mathbf{x}, \psi_{1,0,\ldots,0} \rangle = C_n \mathbf{u}_1 \tag{23}$$

$$\langle \delta\mathbf{x}, \psi_{0,1,\ldots,0} \rangle = C_n \mathbf{u}_2 \tag{24}$$

$$\cdots \tag{25}$$

$$\langle \delta\mathbf{x}, \psi_{0,0,\ldots,1} \rangle = C_n \mathbf{u}_n \tag{26}$$

where C_n is a constant depending only on the number n of atoms in the fine-grained model.

By using this decomposition, it is straightforward to obtain an expression for $\mathcal{P}_\tau \delta\mathbf{x}$:

$$\mathcal{P}_\tau \delta\mathbf{x} = e^{\mathcal{L}\tau} \delta\mathbf{x} = \left(\sum_{i=1}^{n} \mathbf{u}_i e^{-\frac{\lambda_i}{\gamma}} \mathbf{u}_i^\top \right) \delta\mathbf{x} = \tag{27}$$

$$= \Omega_\tau \delta\mathbf{x} \tag{28}$$

where we have defined the matrix:

$$\Omega_\tau = \sum_{i=1}^{n} \mathbf{u}_i e^{-\frac{\lambda_i}{\gamma}} \mathbf{u}_i^\top \tag{29}$$

With this result, we can evaluate the time-lagged covariance matrix as:

$$C_{0\tau} = \langle M\delta\mathbf{x}, M\mathcal{P}_\tau\delta\mathbf{x}\rangle_\mu = \tag{30}$$

$$= M\left(\int \exp\left(-\frac{\delta\mathbf{x}\boldsymbol{\Gamma}\delta\mathbf{x}}{2k_BT}\right)\delta\mathbf{x}\left(\Omega_\tau\delta\mathbf{x}\right)^\top d\mathbf{x}\right)M^\top = \tag{31}$$

$$= M\left(\int \exp\left(-\frac{\delta\mathbf{x}\boldsymbol{\Gamma}\delta\mathbf{x}}{2k_BT}\right)\delta\mathbf{x}\delta\mathbf{x}^\top\right)\Omega_\tau M^\top d\mathbf{x} = \tag{32}$$

$$= QM\boldsymbol{\Gamma}^{-1}\Omega_\tau M^\top Q \tag{33}$$

By putting everything together we have an analytical expression for the VAMP score:

$$\text{VAMP score} = \text{trace}\left(C_{00}^{-1}C_{0\tau}\right) = \text{trace}\left(KC_{0\tau}\right) = \tag{34}$$

$$= \text{trace}\left(\left(QM\boldsymbol{\Gamma}^{-1}MQ\right)^{-1}QM\boldsymbol{\Gamma}^{-1}\Omega_\tau M^\top Q\right) \tag{35}$$

Numerical VAMP Score

The VAMP score can be also directly numerically from the simulated trajectories.[8] By using the definition (Eqn. 3) and 4 and 5) and approximating the ensemble averages with time averages with the time index $t = 1, \ldots, T$ along a long trajectory (or set of trajectories), and symmetrizing to impose detailed balance, we have:

$$C_{00} = \frac{1}{2(T-\tau)}\left(\sum_{t=1}^{T-\tau}\mathbf{X}_t\mathbf{X}_t^\top + \sum_{t=\tau}^{T}\mathbf{X}_t\mathbf{X}_t^\top\right) \tag{36}$$

$$C_{0\tau} = \frac{1}{2(T-\tau)}\left(\sum_{t=1}^{T-\tau}\mathbf{X}_t\mathbf{X}_{t+\tau}^\top + \sum_{t=\tau}^{T}\mathbf{X}_{t-\tau}\mathbf{X}_t^\top\right) \tag{37}$$

where $\mathbf{X}_t$ ($\mathbf{X}_{t+\tau}$) is the vector containing the values of all the selected features (e.g., Cartesian coordinates, or other observables of the CG model) at time t ($t+\tau$). In practice, the numerical evaluation of these matrices and of the VAMP score can be obtained via a standard linear algebra python package.

While the features $\mathbf{X}_t$ chosen for the harmonic systems are the displacements in the Cartesian coordinates and the covariant matrices can be evaluated analytically (see section above), the features for the model protein are the inter-bead distance as calculations with internal coordinates are numerically more stable.

For the harmonic systems, we verified that numerical and analytical calculations yield the same results within numerical precision.

Comparison with the results of Foley, *et al.*[9]

As mentioned in the main text, in ref.[9] Foley *et al.* define a valid mapping as a partitioning of all the 40 C_α's of protein 2ERL into $N = 2, 4, 5, 8, 10, 20$ disjoint groups of an equal number of $40/N$ C_α atoms. They require the C_α atoms belonging to the same CG bead to be connected in the GNM but not necessarily to be subsequent along the sequence. In contrast to ref.,[9] in the present work we require the CG beads to be formed by groups of subsequent atoms, while we also consider partitions of atoms into groups of different sizes, ranging from 1 to $40 - (N - 1)$. In this section, we compare the results presented in the main text with those obtained with the mapping definition from ref.[9]

In Fig. S5B the values of the Vibrational Power are plotted vs. the values of the VAMP score for a sample of the CG maps defined according to the criterion of ref.,[9] when coarse-graining the GNM of protein 2ERL into 4 CG beads. The same data are also shown in Fig. S5A, where they are compared to the values corresponding to all the possible CG maps defined by our mapping criterion (analogous to Fig. 3B in the main text). This comparison shows that the values of the Vibrational Power corresponding to all the CG maps generated with our mapping span a much larger range than the values associated with the mappings adopted by Foley *et al.*[9] Additionally, in the latter case, the VAMP score and Vibrational Power are highly correlated. However, the maps corresponding to the maximum Vibrational Power and the maximum VAMP score do not coincide, even if they are both very close to

the block map (in agreement with what was reported in ref.[9]). In our mapping, by allowing the beads to contain a variable number of atoms, we obtain CG maps with a significantly broader range of values for the Vibrational Power, even if the mapping choice adopted by Foley *et al.*[9] produces a much larger number of possible maps ($\sim 10^{21}$ for $N = 4$ beads).

References

(1) Mendels, D.; de Pablo, J. J. Collective Variables for Free Energy Surface Tailoring: Understanding and Modifying Functionality in Systems Dominated by Rare Events. *J. Phys. Chem. Lett.* **2022**, *13*, 2830–2837.

(2) Eastman, P.; Swails, J.; Chodera, J. D.; McGibbon, R. T.; Zhao, Y.; Beauchamp, K. A.; Wang, L.-P.; Simmonett, A. C.; Harrigan, M. P.; Stern, C. D.; Wiewiora, R. P.; Brooks, B. R.; Pande, V. S. OpenMM 7: Rapid development of high performance algorithms for molecular dynamics. *PLoS Comput. Biol.* **2017**, *13*, e1005659.

(3) Molgedey, L.; Schuster, H. G. Separation of a mixture of independent signals using time delayed correlations. *Phys. Rev. Lett.* **1994**, *72*, 3634–3637.

(4) Pérez-Hernández, G.; Paul, F.; Giorgino, T.; De Fabritiis, G.; Noé, F. Identification of slow molecular order parameters for Markov model construction. *J. Chem. Phys.* **2013**, *139*, 015102.

(5) Schütte, C.; Fischer, A.; Huisinga, W.; Deuflhard, P. A Direct Approach to Conformational Dynamics based on Hybrid Monte Carlo. *J. Comput. Phys.* **1999**, *151*, 146–168.

(6) Foley, T. T.; Shell, M. S.; Noid, W. G. The impact of resolution upon entropy and information in coarse-grained models. *J. Chem. Phys.* **2015**, *143*, 243104.

(7) Nüske, F. A variational approach for conformation dynamics. M.Sc. thesis, Freie Universität Berlin, Berlin, 2012.

(8) Wu, H.; Noé, F. Variational Approach for Learning Markov Processes from Time Series Data. *J. Nonlinear Sci.* **2020**, *30*, 23–66.

(9) Foley, T. T.; Kidder, K. M.; Shell, M. S.; Noid, W. G. Exploring the landscape of model representations. *Proc. Natl. Acad. Sci. U.S.A.* **2020**, *117*, 24061–24068.

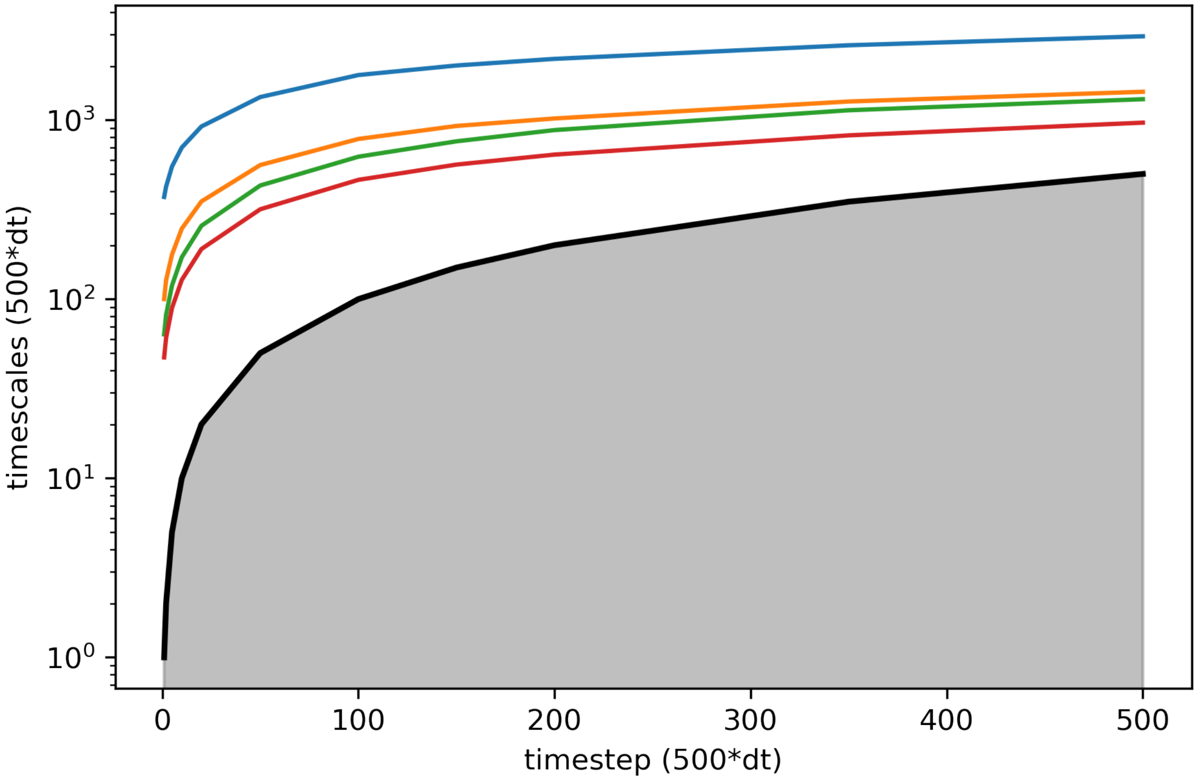

Figure S1: The implied timescale of the model protein for the 4 leading processes. The implied timescales of the system refer to the intrinsic relaxation times of the system. The shaded grey area indicates the region where Markov processes can not be resolved. Note the simulation data has been strided by a factor of 500 and data is shown on a log-log scale. From this we select a lagtime of 100 to compute the VAMP score for this system.

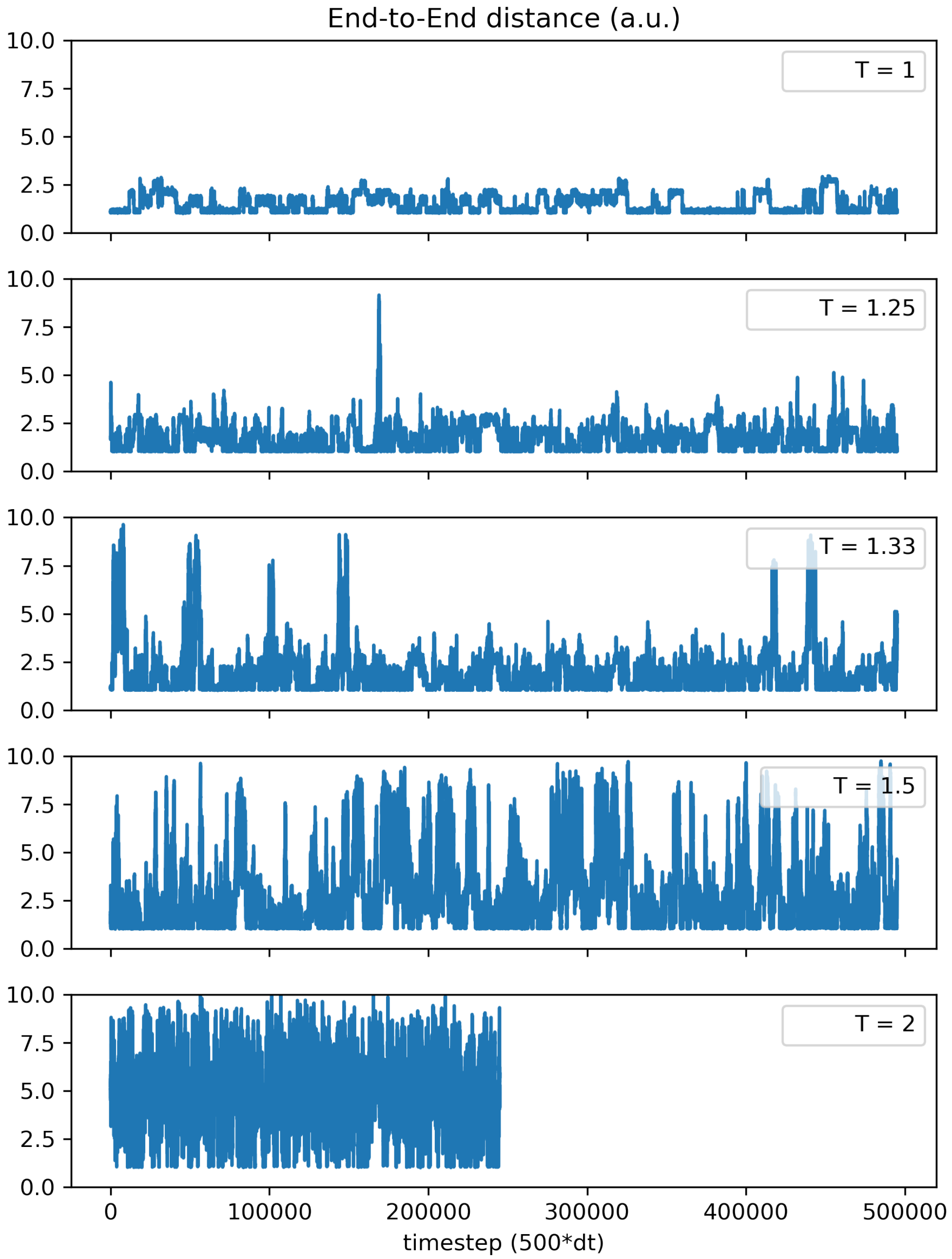

Figure S2: End to End distance of the model protein at 5 different temperatures, indicated by the y-axis label, that increase progressively from $k_BT = 1$ to $k_BT = 2$. For the top row at $k_BT = 1$ we see no unfolding events while the bottom row at $k_BT = 2$ has no apparent preference towards the folded state. The x-axis indicates the timestep strided again by 500. The temperature of the system corresponding to one that has a few but not too many transitions between the folded and unfolded state is suitable for our study.

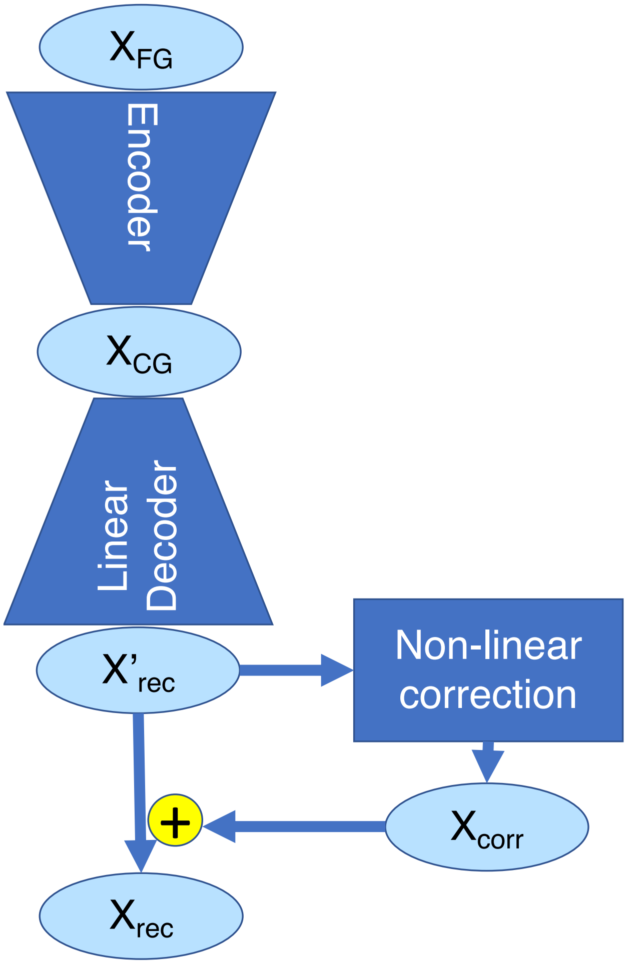

Figure S3: The architecture of the autoencoder model to evaluate the reconstruction error. The decoder consists of one linear and one non-linear module. The two modules are trained separately, one after the other. In the figure, X'_{rec} indicates the FG coordinates as initially reconstructed by the linear decoder, and X_{corr} is the correction provided by the non-linear component of the decoder.

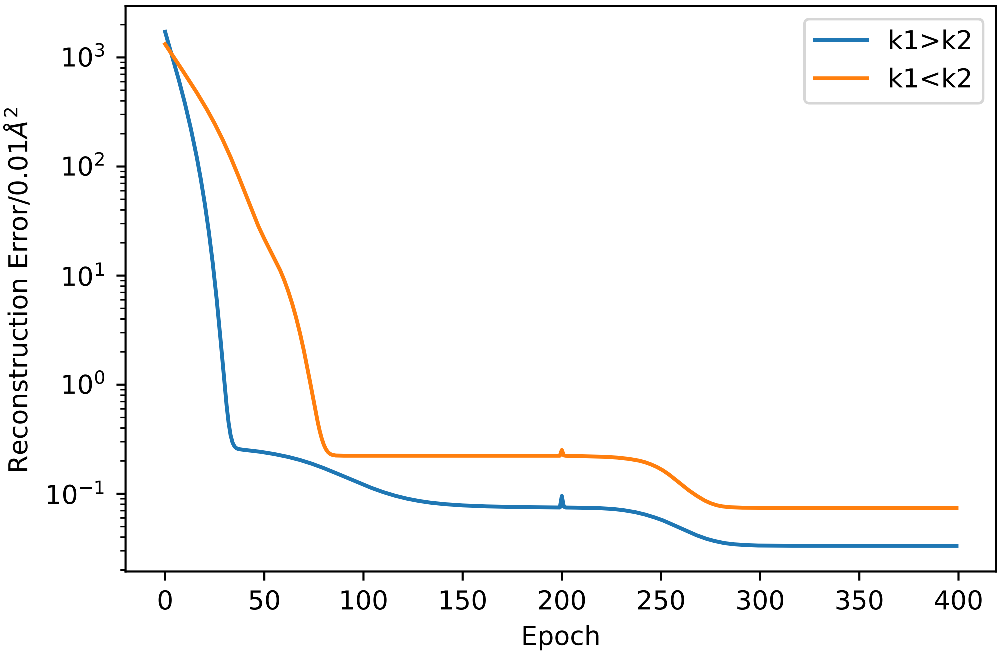

(a) 4-bead System

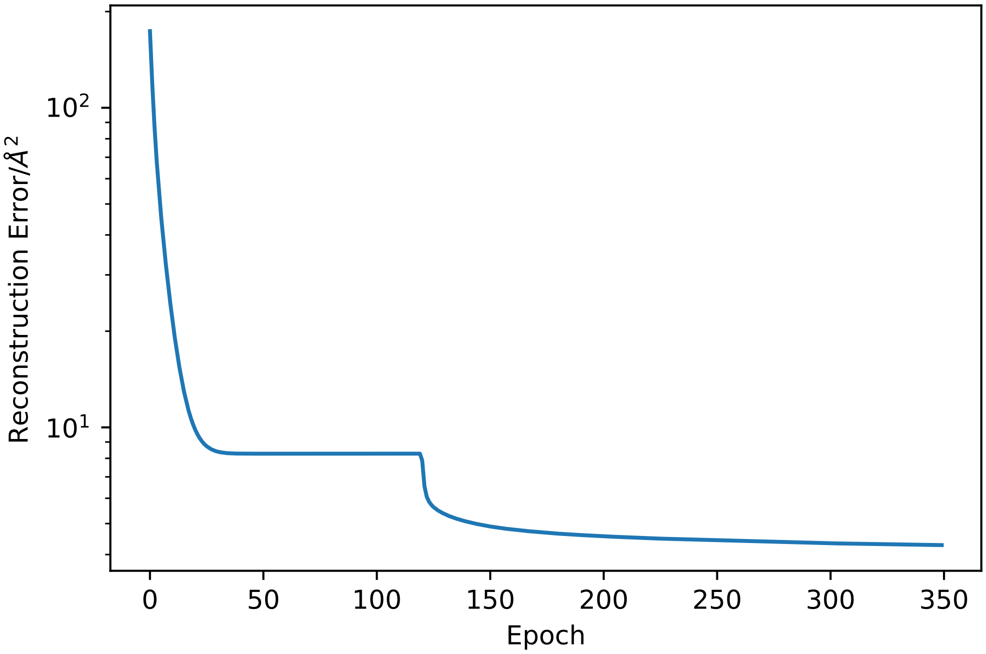

(b) Model Protein

Figure S4: Validation loss corresponding to the training of the reconstruction autoencoder for the CG mapping with minimum RE, for the 4-bead harmonic model (a) and model protein system (b).

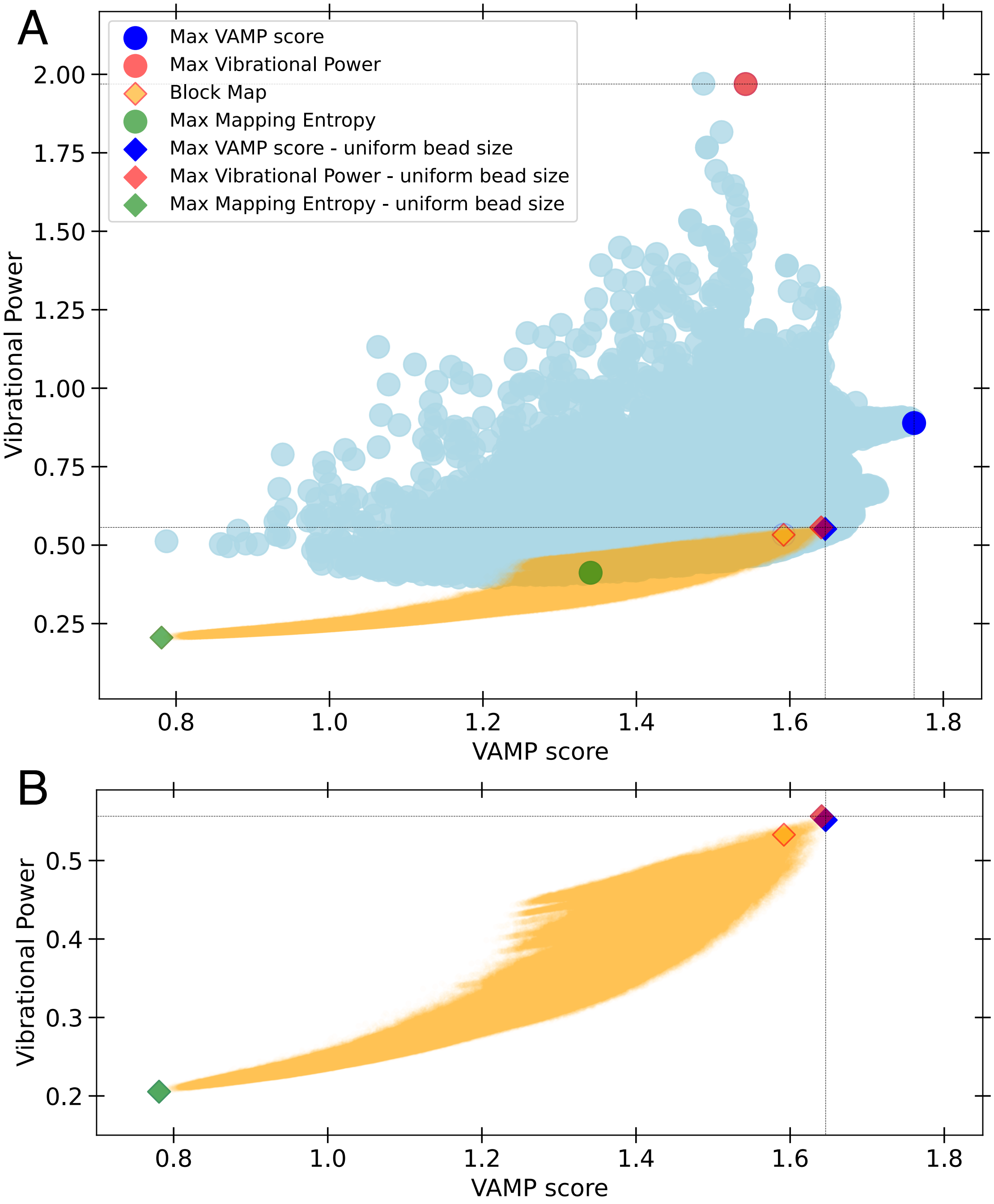

Figure S5: A) Comparison of the Vibrational Power and VAMP score calculated for all possible CG maps defined by our mapping criterion (light blue dots), and for a sample of CG maps defined by the criterion of ref.[9] (orange dots), for the GNM of protein 2ERL coarse-grained into 4 beads. The values corresponding to the maximum VAMP score, maximum Vibrational Power, and Maximum Mapping Entropy are highlighted by blue, red, and green dots, respectively for our mapping choice, and by blue, red, and green diamonds for the mapping choice of ref.[9] The values corresponding to the block map (that is, the CG map where each of the 4 beads contains 10 consecutive atoms) are marked by an orange diamond. B) The same data from A) for a sample of CG maps defined by the criterion of ref.[9] are plotted in a zoomed-in range of Vibrational Power to show the structure of the data.
